# Supplementary material for: Pleurotus Ostreatus Ameliorates Obesity by Modulating the Gut Microbiota in Obese Mice Induced by High-Fat Diet
Source: Nutrients. 2022 Apr 29;14(9):1868. doi: 10.3390/nu14091868 (PMC9103077; doi:10.3390/nu14091868)
Supplement: Supplementary file 1 [file nutrients-14-01868-s001.zip › nutrients-1667960-supplementary.pdf]

Supplementary Table S1. Composition of diets.

	LFD		HFD	
	**Mass percentage (%)**	**Percentage of energy (%)**	**Mass percentage (%)**	**Percentage of energy (%)**
Protein	19.2	20	26	20
Carbohydrate	67.3	70	26	20
Fat	4.3	10	35	60
total		100		100
kcal/g	3.85		5.24	

Supplementary Table S2. Composition of *Pleurotus Ostreatus* powder (value = mean ± SD).

**Compositions**	**%**
Water	8.54 ± 0.14
Ash	4.90 ± 0.03
Carbohydrate	63.44 ± 0.05
Fat	1.81 ± 0.04
Protein	19.07 ± 0.30
Dietary fiber	2.25 ± 0.04
Polysaccharide	3.58 ± 0.29

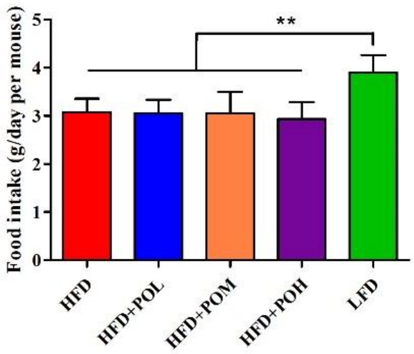

Supplementary Figure S1. Food intake.

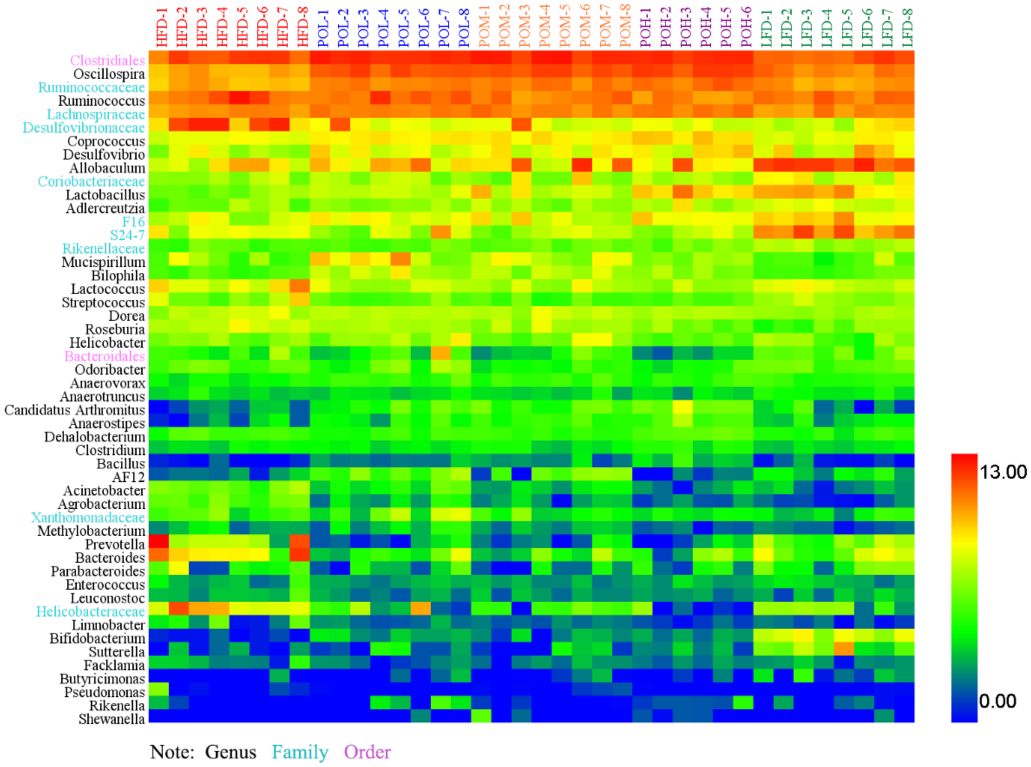

Supplementary Figure S2. The gut microbiota profile of every mouse.
